# A method for selecting reference beam model of VMAT plans with three 6MV beam-matched linear accelerators during radiation oncology

**DOI:** 10.1038/s41598-023-36930-7

**Published:** 2023-06-22

**Authors:** Yi Li, Wenjing Wu, Wei Yuan, Linyan Chai, Fengwen Tang, Ruixin He, Yongkai Lu, Yuemei Zhang, Yongkai Lu, Long Wang, Mengfei Wang, Xiaozhi Zhang, Long Zhang

**Affiliations:** 1 Department of Radiation Oncology, The First Affiliated Hospital of Xi’an Jiaotong University, Xi’an, 710061 China; 2 Department of Radiological Health, Xi’an Center for Disease Control and Prevention, Xi’an, 710054 China

**Keywords:** Radiotherapy, Biomedical engineering, Experimental nuclear physics

## Abstract

Our objective was to provide a method for selecting reference beam model and evaluating the dosimetric accuracy of volumetric modulated arc therapy (VMAT) plans delivered on three Elekta beam-matched linacs during radiation oncology. Beam data was measured on three beam-matched linacs including Synergy1, Synergy2 and VersaHD. For eighteen lung and esophagus cases, fifty-four plans were generated using VMAT technique with three linac beam models respectively for point dose measurement and three-dimensional dose measurement. Each VMAT plan was executed sequentially on three linacs respectively. Measurement results were compared with treatment planning system (TPS) calculation results for all VMAT plans. Among three beam-matched linacs, discrepancy in beam output factor, percentage depth dose at 5 cm, 10 cm, 20 cm depth and MLC leaf offset are all within 1% except 20 × 20 cm^2^ and 30 × 30 cm^2^ field sizes, and discrepancy in beam profile is all within 2%. With comparison between measurement result and TPS calculation result, the absolute dose deviations are within the range of ± 3%, and the gamma passing rates are all over 95% for all VMAT plans, which are within the tolerance of clinical acceptability. Compared with all plans delivered on Synegy1 and VersaHD, the point dose discrepancy between measured results and TPS calculated results for plans delivered on Synergy2 is smallest, and the gamma passing rate between measured results and TPS calculated results for plans delivered on Synergy2 is highest. The beam-matched linacs demonstrate good agreement between measurement result and TPS calculation result for VMAT plans. The method can be used for selecting reference beam model for VMAT plans.

The success of curative radiation therapy largely depends on the ability of the treatment device to delivery properly the prescribed dose to the entire tumor volume within a narrow tolerance. A 5% discrepancy in the delivered dose may result in changes in the order of 10–20% in tumor control probability and 20–30% in normal tissue complication probability^1,2^. Therefore, it is necessary to reduce uncertainty at every stage of treatment. However, treatment may be affected by sudden breakdown of any linac, unexpected high patient load, or other reasons^3^. It can be greatly improved if several linear accelerators (linacs) can be beam-matched and patients can be treated using any linac without the need to adjust the treatment plans^4–6^.

Having beam-matched linear accelerators can not only increase the flexibility in patient treatment but also reduce the social and economic effects caused by machine down time^7^. Several studies have reported beam-matching results and beam data reproducibility for Varian, Elekta, and Siemens linacs^4,8–10^. Xu et al.^4^ have evaluated dose delivery accuracy of volumetric modulated arc therapy (VMAT) plans on three beam-matched linacs when selecting a linac as the reference for tuning of beams of other linacs. However, which linac model suited as the standard beam model is not available for all three beam-matched linacs in previous literatures. In our study, we provide a method for selecting reference beam model and evaluating the dosimetric accuracy of VMAT plans delivered on three Elekta beam-matched linacs.

## Methods

### Beam data acquisition for three beam-matched Elekta linacs

Three Elekta beam-matched linacs were installed in our department including Synergy1, Synergy2 and VersaHD, which had been equipped with Agility heads (80 MLC leaf pairs of 5 mm leaf width). The condition of three linear accelerators is shown in Table 1. Percentage depth dose (PDD), beam profiles, output factors (OF), and MLC leaf offset were measured on all three beam-matched linacs. All measurements were conducted using the IBA Blue Phantom scanning phantom system (IBA dosimetry, Germany). PDDs at 5 cm depth (PDD5), 10 cm depth (PDD10) and 20 cm depth (PDD20) were measured for beam of 10 × 10 cm^2^ field size. According to the recommendation by American Association of Physicists in Medicine (AAPM) Task Group 101^11^, IBA CC13 ion chamber with 0.13 cm^3^ cavity volume was used to measure PDD, beam profile, MLC leaf offset. Moreover, CC13 was used to measure OF for field size larger than or equal to 5 × 5 cm^2^, while IBA CC01 ion chamber of 0.01 m^3^ cavity volume was used to measure OF for field size less than 5 × 5 cm^2^.

**Table 1:** The condition of the three linear accelerators.

Maximum gantry rotation speed	1 revolution per minute	Maximum jaw speed	3.2 cm/s
Maximum MLC leaf speed	3.2 cm/s	Maximum gantry MU delivery	20 MU/degree
Minimum gantry MU delivery	0.3 MU/degree	Minimum MLC leaf MU delivery	0.3 MU/cm
Maximum dose rate	600 MU/min	Maximum leaf travel distance per gantry degree	
Maximum arc delivery time			

### Evaluation method of dosimetric accuracy

Eighteen lung and esophagus cases were prescribed with 60 Gy in 30 fractions. All VMAT plans were generated in Monaco TPS using beam model from Synergy1, Synergy2, VersaHD and were named as plans1, plans2, plans3, respectively. Lung VMAT plans were generated with two partial arcs and 6MV photon beams, while esophagus plans were generated with three partial arcs and 6MV photon beams. Minimum leaf width was 7 mm and the calculation grid was 2 mm for VMAT plan setting. In addition, Monte Carlo method was adopted for VMAT planning with statistical uncertainty less than 1%. All plans were delivered on three beam-matched linacs respectively.

**Figure 1:**
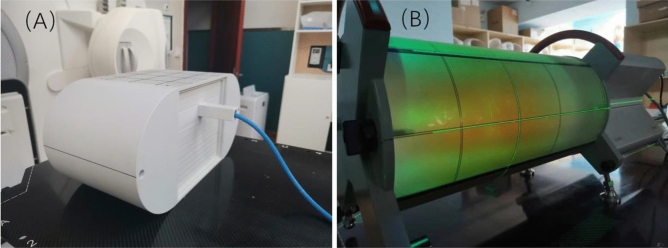
The point dose measurement with (**A**) IMRT phantom and 3D relative dose distribution measurement with (**B**) Delta4 cylindrical diode array system.

All VMAT plans were measured using PTW ion chamber and Delta4 cylindrical diode array system for absolute point dose measurement and three-dimensional dose measurement respectively. The PTW Farmer Chamber of 0.6 cm^2^ was placed in the IMRT phantom (IBA, German) for absolute point does measurement as shown in Fig. 1. Ion chamber measurement results and Delta4 measurement results were compared with the TPS calculated point dose in IMRT phantom and three-dimension (3D) dose in Delta4 phantom with 3%/2 mm gamma criteria, respectively. The tolerance limit in point dose discrepancy and gamma-passing rate of 3D dose is less than 3% and greater than 95%, respectively^12^. Discrepancy in point dose measurement and gamma-passing rate of 3D dose among three linacs were analyzed to select the reference beam model and evaluate dosimetric accuracy of VMAT plans delivered on three Elekta beam-matched linacs.

### Statistical analysis

All statistical analysis was performed by SPSS Statistics V22.0 software (IBM Corp., Armonk, NY). Quantitative data was expressed as mean ± standard deviation (SD). Paired T test was used to analyse point dose discrepancy and 3D dose discrepancy among three beam-matched linacs. Discrepancy was considered significant for *P* < 0.05.

## Results

### Beam matched results

The variation in repeated OF measurements using ion chamber for three beam-matched linacs are all within 1% except 20 × 20 cm^2^ and 30 × 30 cm^2^ field sizes as shown in Table 2. The maximum discrepancy in OF is 1.3%, which is the discrepancy in OF of 20 × 20 cm^2^ field size between Synergy1 and Synergy2. Discrepancy in PDD5, PDD10, PDD20 and MLC leaf offset are all equal or less than 0.5% as shown in Table 3. For beam profiles from 3 × 3 cm^2^ to 40 × 40 cm^2^ field sizes at 10 cm depth, any point dose of a linac within the region covering 80% of full width at half maximum is within a 2% discrepancy compared with the same points from profiles of other beam-matched linacs.

**Table 2:** Output factors for 6MV photon beam of different field sizes for three beam-matched linacs.

Field size (cm × cm)	Synergy1	Synergy2	VersaHD	Maximal discrepancy (%)
2 × 2	0.796	0.798	0.792	0.6
3 × 3	0.844	0.843	0.839	0.5
4 × 4	0.877	0.877	0.876	0.1
5 × 5	0.906	0.906	0.899	0.7
7 × 7	0.952	0.951	0.946	0.6
10 × 10	1	1	1	0.0
15 × 15	1.058	1.055	1.062	0.7
20 × 20	1.096	1.092	1.105	1.3
30 × 30	1.145	1.135	1.146	1.1
40 × 40	1.164	1.158	1.168	1.0

**Table 3:** Discrepancy in PDD for 10 × 10 cm^2^ field size and MLC leaf offset among three beam-matched linacs.

6MV	Synergy1	Synergy2	VersaHD	Maximal discrepancy (%)
PDD5 (%)	86.71	86.58	86.5	0.20
PDD10 (%)	67.59	67.75	67.20	0.50
PDD20 (%)	39.66	39.59	39.38	0.30
MLC leaf offset (mm)	0.15	0.10	0.00	0.15

### Point dose measurement results

**Figure 2:**
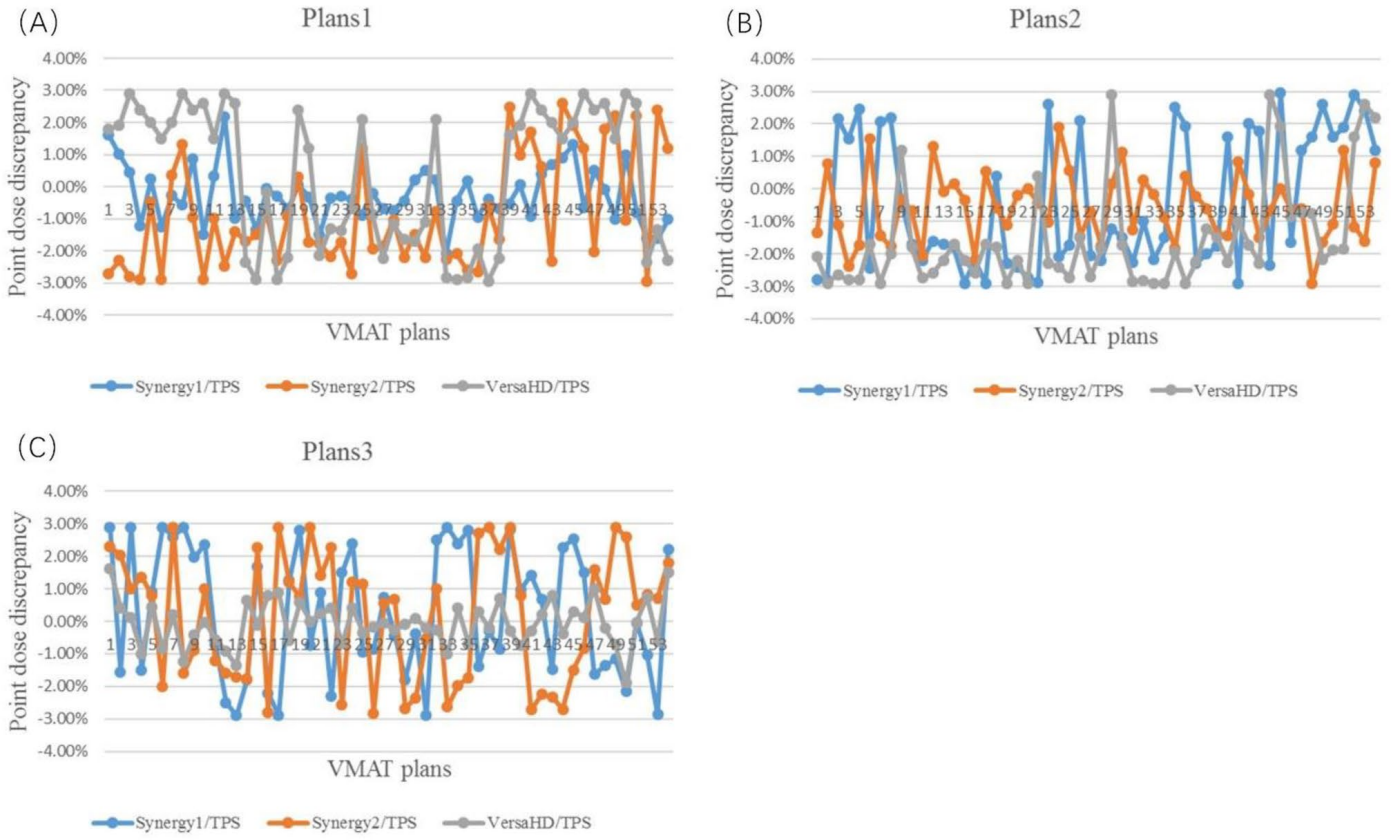
The dose discrepancy distribution between ionization chamber measurement and TPS calculation. (**A**) Point dose discrepancy of plans1, (**B**) point dose discrepancy of plans2, (**C**) point dose discrepancy of plans3).

For all VMAT plans, the point dose discrepancy between chamber measurement and TPS calculation is demonstrated in Fig. 2 and Table 4. The point dose discrepancy is less than 3%, which is within the tolerance recommend by the AAPM 218 reports^12^. For plans1, the variance range of the point dose discrepancy is from -2.96% to 2.90%. The point dose discrepancy for plans1 delivered on Synergy1 is smaller than that delivered on Synergy2 and VersaHD (t = 11.145, 13.532; *P* < 0.05). For plans2, the variance range of the point dose discrepancy is from − 2.90 to 2.90%. The point dose discrepancy for plans2 delivered on Synergy2 is smaller than that delivered on Synergy1 and VersaHD (t = 8.703, 8.816; *P* < 0.05). For plans3, the variance range of the point dose discrepancy is from − 2.92 to 2.95%. The point dose discrepancy for plan3 delivered on VersaHD is smaller than that delivered on Synergy1 and Synergy2 (t = 11.469, 10.594; *P* < 0.05). In sum, the point dose discrepancy of VMAT plan delivered on the linac with same plan model is lowest. Moreover, the point dose discrepancy of plans2 is smallest among all 3D dose distribution results.

**Table 4:** The point dose discrepancy analysis between ionization chamber measurement and TPS calculation (%, Mean ± SD).

	Plans1	Plans2	Plans3
Discrepancy_Synergy1 versus TPS_	− 0.27 ± 0.87	0.24 ± 1.98	− 0.57 ± 1.07
Discrepancy_Syergy2 versus TPS_	− 0.88 ± 1.74	0.15 ± 1.97	− 0.42 ± 2.10
Discrepancy_VersaHD versus TPS_	0.37 ± 2.18	− 0.09 ± 0.66	− 1.55 ± 1.62
Discrepancy_Synergy2 versus Synergy1_	− 0.61 ± 1.96	− 0.10 ± 2.89	− 1.13 ± 2.42
Discrepancy_VersaHD versus Synergy1_	0.64 ± 1.99	− 0.34 ± 2.07	− 0.15 ± 2.68
Discrepancy_VersaHD versus Synergy2_	1.25 ± 2.26	− 0.24 ± 1.94	− 0.97 ± 1.88

### Three-dimensional dose measurement results

**Figure 3:**
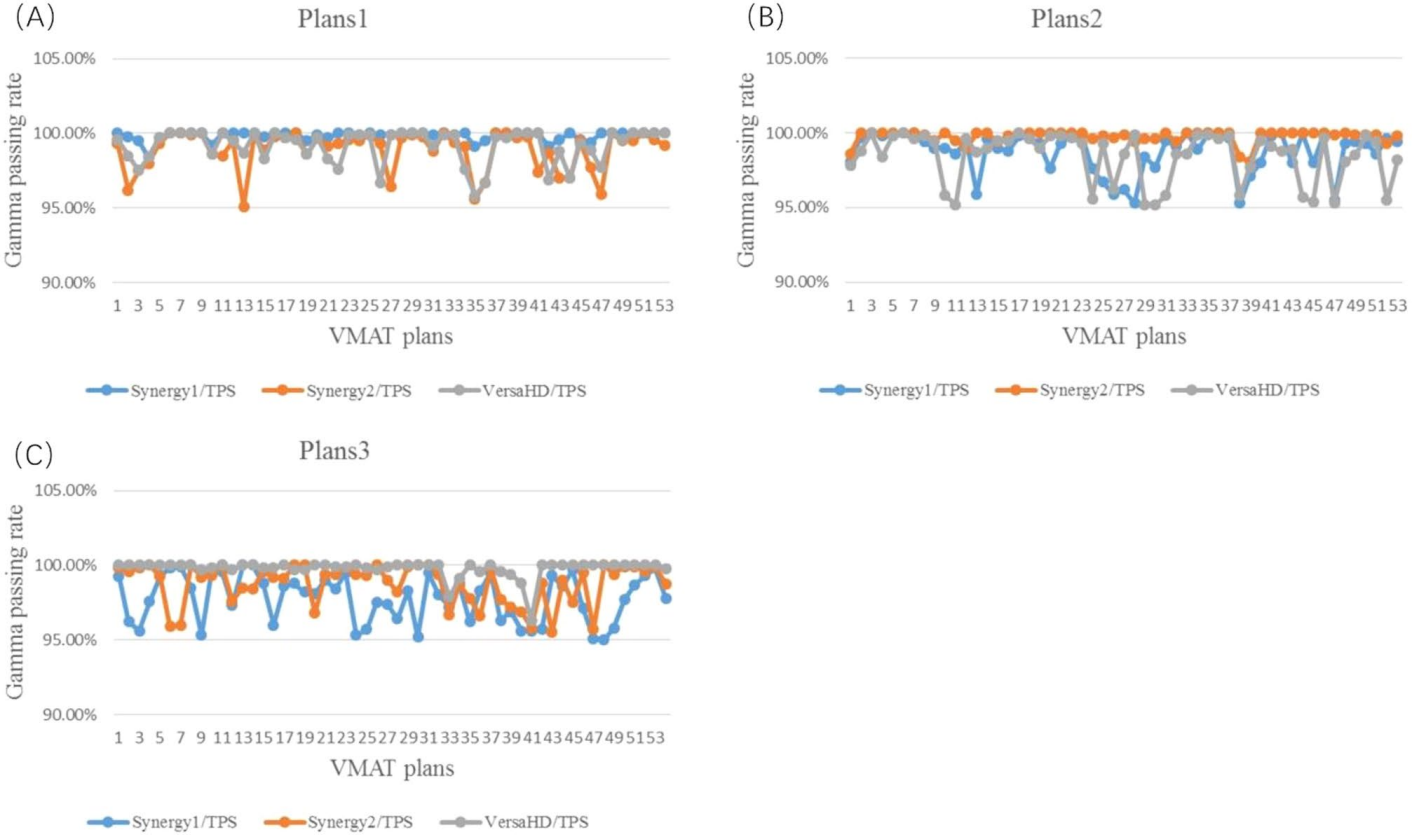
Gamma-passing rate of three-dimensional dose measured by Delta4 (**A**) the gamma-passing rate for plans1 delivered on three accelerators, (**B**) the gamma-passing rate for plans2 delivered on three accelerators, (**C**) the gamma-passing rate for plans3 delivered on three accelerators.

For all VMAT plans, the 3D dose discrepancy between chamber measurement and TPS calculation is demonstrated in Fig. 3 and Table 5. The gamma-passing rate of 3D dose is greater than 95%, which is within the tolerance recommend by the AAPM 218 reports^12^. For plans1, the variance range of gamma-passing rate is from 95.10 to 100.00%. The gamma passing rate for plans1 delivered on Synergy1 is higher than that delivered on Synergy2 and VersaHD (t = 6.312, 6.169; *P* < 0.05). For plans2, the variance range of gamma-passing rate is from 95.20 to 100.00%. The gamma-passing rate for plans2 delivered on Synergy2 is higher than that delivered on Synergy1 and VersaHD (t = 5.924, 6.286; *P* < 0.05). For plans3, the variance range of the gamma-passing rate is from 95.00 to 100.00%. The gamma-passing rate for plan3 delivered on VersaHD is higher than that delivered on Synergy1 and Synergy2 (t = 9.223, 5.982; *P* < 0.05). In sum, the gamma-passing rate of VMAT plan delivered on the linac with same plan model is highest. Moreover, the gamma-passing rate of plans2 is smallest among all 3D dose distribution results.

**Table 5:** The gamma-passing rate analysis of three-dimensional dose distribution measured by Delta4 (%, Mean ± SD).

	Plans1	Plans2	Plans3
γ_Synergy1 versus TPS_	99.84 ± 0.31	98.75 ± 1.38	97.79 ± 1.61
γ_Synergy2 versus TPS_	98.89 ± 1.32	99.77 ± 0.42	98.75 ± 1.37
γ_VersaHD versus TPS_	99.16 ± 1.12	99.41 ± 1.66	99.78 ± 0.60
Discrepancy_Synergy2 versus Synergy1_	− 0.95 ± 1.23	1.02 ± 1.26	0.96 ± 2.04
Discrepancy_VersaHD versus Synergy1_	− 0.68 ± 0.99	− 0.34 ± 1.74	1.99 ± 1.57
Discrepancy_VersaHD versus Synergy2_	0.27 ± 1.10	− 1.36 ± 1.59	1.03 ± 1.25

## Discussion

Many dosimetry factors, such as OF^13^, PDD^14^, dose profile^15^, MLC position^16^, can be consistence across different linacs by beam-matched method. It is possible for interchanging patients for beam-matched linear accelerators from the same vendor, which would make a significant contribution to the efficiency of treatment. In order to select reference linac model for VMAT plan, we analyzed discrepancy in dosimetric beam data collected during commissioning and evaluated dosimetric accuracy of point dose and 3D dose distribution among three Elekta beam-matched linacs.

The average discrepancies in OF, PDD and MLC leaf offset among three beam-matched linacs are equal or less than 1% except of 20 × 20 cm^2^ field size and 30 × 30 cm^2^ field size, which indicates that these linacs are beam-matched well. Xu et al. reported that the average discrepancy in the measurement of OF and PDD among three beam-matched Elekta linacs were less than 1%^4^. Bhangle et al. reported that all evaluated dosimetric factors from two Siemens linacs were within 1%. Our evaluated dosimetric results are the same with those of the previous studies^9^.

Verification of dose delivery for the dose calculation is essential for beam-matched linacs. In this study, deviation of all point dose measurements fells within ± 3%, the gamma-passing rates of 3D dose distribution are greater than 95%, which is similar with the results by Ashokkumar^17^. Therefore, the dosimetric analysis of thorax VMAT plans swapped between three machines are all within clinical acceptable limits in this study. These results support the swapping of patient across beam-matched linear accelerators in busy clinical environment without replanning of VMAT plans. However, Care must be taken to ensure the verification of beam matching prior to implementation.

Among all VMAT plans with one machine model, the point dose discrepancy between measured results and TPS calculated results for VMAT plans delivered on the machine with same plan model is smallest, and the gamma passing rate for VMAT plans delivered on the same machine is highest. Moreover, while interchanging the plans among three Elekta machines, compared with plans1 and plans3, the gamma-passing rate of plans2 is highest among all 3D dose distribution results. Therefore, Synergy2 model can be used as the reference beam model for VMAT plans.

Among all dosimetry factors, MLC position is an important factor affecting passing rate and beam-matched results. Oliver et al. reported that the maximum variation of dose delivery due to random MLC positional errors of 1 mm is around 1.21%^18^. Moreover, many factors including grieshoch, burn-in, abrasion can affect MLC position accuracy. There are two methods including MLC calibration^19^ in linac and MLC leaf offset setting^20^ in TPS to solve the problem. This also indicates that, if beam match is adopted, a weekly or monthly quality assurance (QA) of VMAT plan across all linacs is required on all linacs to ensure that MLC calibration or MLC leaf offset has not drifted.

## Conclusion

This study provides a selected method of reference beam model and evaluate the dosimetric accuracy of VMAT plans delivered on three Elekta beam-matched linacs. We concluded that the beam-matched linacs demonstrated good agreement between measurement result and TPS calculation result for VMAT plans. Synergy2 model can be used as reference beam model for VMAT plans. However, many factors can affect beam-matched results. A periodic QA should be taken to ensure the accuracy of dose delivery on beam-matched linacs.

## Data Availability

The datasets used and analysed during the current study available from the corresponding author on reasonable request.
